# The p38alpha mitogen-activated protein kinase limits the CNS proinflammatory cytokine response to systemic lipopolysaccharide, potentially through an IL-10 dependent mechanism

**DOI:** 10.1186/s12974-014-0175-6

**Published:** 2014-10-10

**Authors:** Adam D Bachstetter, Bin Xing, Linda J Van Eldik

**Affiliations:** Sanders-Brown Center on Aging, University of Kentucky, 800 S. Limestone Street, Lexington, KY 40536 USA; Department of Anatomy and Neurobiology, University of Kentucky, 800 S. Limestone Street, Lexington, KY 40536 USA; Current Affiliation: VA Pittsburgh Healthcare System, 3501 Fifth Avenue, Pittsburgh, PA 15260 USA

**Keywords:** cytokines, glia, interleukin, neuroinflammation, signal transduction, tumor necrosis factor alpha

## Abstract

**Background:**

The p38α mitogen-activated protein kinase (MAPK) is a well-characterized intracellular kinase involved in the overproduction of proinflammatory cytokines from glia. As such, p38α appears to be a promising therapeutic target for neurodegenerative diseases associated with neuroinflammation. However, the *in vivo* role of p38α in cytokine production in the CNS is poorly defined, and prior work suggests that p38α may be affecting a yet to be identified negative feedback mechanism that limits the acute, injury-induced proinflammatory cytokine surge in the CNS.

**Methods:**

To attempt to define this negative feedback mechanism, we used two *in vitro* and two *in vivo* models of neuroinflammation in a mouse where p38α is deficient in cells of the myeloid lineage.

**Results:**

We found that p38α in myeloid cells has an important role in limiting amplitude of the acute proinflammatory cytokine response to a systemic inflammatory challenge. Moreover, we identified IL-10 as a potential negative feedback mechanism regulated by p38α.

**Conclusions:**

Our data suggest that p38α regulates a proper balance between the pro- and anti-inflammatory cytokine responses to systemic inflammation, and that if circulating IL-10 levels are not elevated to counter-balance the increased systemic proinflammatory responses, the spread of the inflammatory response from the periphery to the CNS is exaggerated.

## Introduction

Inflammation is a complex response that requires tight regulatory control of its initiation and resolution. In the central nervous system (CNS), neuroinflammation has gained increasing support as a contributing mechanism in a number of neurodegenerative diseases [1,2], including Alzheimer’s disease (AD) [3] and Parkinson’s disease [4], as well as acute neurodegenerative conditions such as traumatic brain injury (TBI) [5], spinal cord injury [6] and stroke [7]. As one aspect of neuroinflammation, proinflammatory cytokines have been linked to tissue injury and disease processes [8,9]. For example, overproduction of IL-1β by activated microglia can induce pathological tau phosphorylation and a decline in synaptophysin levels in neurons [10,11]. Cytokine production is regulated at multiple levels; however, one intracellular mechanism involved in upregulated cytokine production is the p38 mitogen-activated protein kinase (MAPK). The p38 MAPK family consists of at least four isoforms (p38α,β,δ,γ), which are encoded by separate genes, expressed in different tissues and cell types, and are often functionally distinct [12]. Mechanistically, the p38 MAPKs phosphorylate protein substrates that are downstream targets involved in the regulation of proinflammatory cytokine gene expression through effects on transcription and translation (for review see: [12]).

Since the discovery of the pyridinyl imidazole class of small molecule anti-inflammatory compounds that inhibit the activity of p38 (commonly known as ‘SB’ compounds) [13], extensive resources have been allocated to the development and refinement of p38 inhibitors for the treatment of peripheral inflammatory diseases [14,15]. A few studies have used small molecule p38 inhibitors *in vivo* to demonstrate that pharmacological inhibition of p38α is neuroprotective in animal models of CNS disorders, such as AD [16,17], global and focal ischemia [18-21], neuropathic pain [22], and seizures [23]. However, human clinical trial experience with p38α inhibitors has yielded mixed results. Published data on the effects of p38 inhibition in cardiovascular disease, chronic obstructive pulmonary disease, and neuropathic pain show encouraging results, whereas trials in rheumatoid arthritis and Crohn’s disease show limited, if any, efficacy (for review see: [24]). These results emphasize the importance of a better understanding of the *in vivo* role of p38α in various inflammatory diseases, especially neuroinflammatory disorders of the CNS.

While p38α appears promising as a therapeutic target for neurodegenerative diseases [17,25,26], more preclinical work is necessary to determine the appropriate disease indications, therapeutic window, and potential side effects of inhibiting the p38α pathway. For example, *in vitro* studies have demonstrated that the p38α isoform contributes to approximately 50% of the IL-1β and TNFα produced by microglia in response to inflammatory stimuli [27]. In contrast, following a diffuse TBI in mice with a genetic knockdown of p38α in cells of the myeloid lineage (p38α^ΔLysM-Cre^ KO mice), an enhanced CNS acute cytokine surge (more IL-1β, IL-6, TNFα) was found at 6 hr post-injury; however by 7 days post-injury, cytokine levels in the p38α^ΔLysM-Cre^ KO mice were below that of the injured wild-type (WT) mice [28]. These results suggest that p38α may be affecting a yet to be identified negative feedback mechanism that limits the acute cytokine surge occurring during the first hours after an *in vivo* insult, which is not evident *in vitro*. The goal of this project was to explore the potential mechanism by which p38α could suppress the acute proinflammatory cytokine response in the CNS. We report here that the CNS proinflammatory response to systemic lipopolysaccharide (LPS) administration is enhanced in the p38α^ΔLysM-Cre^ KO mice compared to WT mice, and that p38α is required by myeloid cells to produce IL-10 in response to LPS. Our results suggest that p38α has a critical role in limiting the spread of the peripheral inflammatory response to the CNS through regulating the production of the anti-inflammatory cytokine IL-10.

## Materials and methods

### Reagents

Lipopolysaccharides (LPS) from *Salmonella enterica serotype typhimurium* (Sigma-Aldrich, St. Louis, MO, USA: Cat. no. L6143-1MG; EU/MG of LPS is 600,000) was prepared in sterile 0.9% sodium chloride that was free of preservatives (Hospira, Inc., Lake Forest, IL: cat. no. NDC 0409-4888-10). Sterile 0.9% sodium chloride was used as the vehicle (veh) control in all experiments.

### Animals

Experiments were conducted in accordance with the principles of animal care and experimentation in the Guide For the Care and Use of Laboratory Animals. The Institutional Animal Care and Use Committee of the University of Kentucky approved the use of animals in this study (protocol #2010-0615). The p38α^ΔLysM-Cre^ KO mice were generated as previously described [29]. The first exon of the p38α gene (*MAPK14*) was flanked by two loxP sites. The p38α floxed mice were maintained as homozygotes: p38α^fl/fl^. To induce cell-specific deletion of p38α MAPK in myeloid cells including macrophages and microglia, the p38α^fl/fl^ mice were crossed with mice that contain Cre driven by the lysozyme promoter. The restricted cell-type expression of the lysozyme promoter [30,31] results in myeloid cell-specific deletion, as we have previously validated [27,28,32]. The mice were backcrossed to generate mice that were null for mouse WT p38α, homozygous for floxed p38α, and hemizyogous for LysM-Cre: p38α^fl/fl;LysM-Cre^. The p38α^fl/fl^ were crossed with the p38α^fl/fl;LysM-Cre^ to generate litters where approximately 50% of the mice are microglia/macrophage conditional p38α KO (p38α^ΔLysM-Cre^ KO) and approximately 50% are p38α^fl/fl^ littermates (used as WT controls). All experiments used both genders of mice at approximately a 50:50 ratio. Genotyping was performed by Transnetyx, Inc (Cordova, TN, USA). On the basis of preliminary data and prior published work [27], we find cytokine levels to be consistently below or at the limit of detection in unstimulated mice. Therefore, to reduce the total number of mice needed for the experiments, the number of mice treated with veh alone was kept to the minimum (n = 2 per group/time point) necessary to confirm there was no elevated basal cytokine response due to experimental manipulation or other idiopathic cause.

### Lipopolysaccharide administration

For intracerebroventricular (ICV) administration of LPS, three to four month old male and female mice were anesthetized with 5% isoflurane prior to stabilizing the head using ear bars in a digital mouse stereotaxic frame (Stoelting Co, Wood Dale, IL, USA). Anesthesia was maintained with continuous inhalation of isoflurane (3.5%, 1 liter/min). A midline incision was made in the scalp to expose the skull. A hole was drilled into the skull over the right lateral ventricle at the following coordinates: AP = -0.5 mm; ML = -1.0 mm. With a 10-μl Hamilton syringe with a blunt 28-gauge needle, veh or LPS was injected at the following coordinates: AP = -0.5 mm; ML = -1.0 mm; DV = -1.8 mm. The Quintessential Stereotaxic Injector (Stoelting Co, Wood Dale, IL, USA) was used to inject 2 μl at a rate of 0.5 μl per min. After injection, the needle was left in place for 2 min before being slowly withdrawn. The incision was closed using staples, and the animal was kept on a heat pad until return of normal activity, at which time the mouse was returned to its home cage. Serum and brain tissues were harvested at 6 hr after treatment. Intraperitoneal (IP) administrations of LPS (5 mg/kg) or veh were done as previously described [27]. Serum and brain tissues were harvested at 1, 6, and 24 hr after treatment.

### Tissue collection and processing

Mice were injected with an overdose of sodium pentobarbital (Pentasol powder; Vibrac Animal Health, Ft Worth, TX, USA: cat. no. NDC-051311-103-25). Blood was collected by cardiac puncture, prior to transcardiac perfusion with ice-cold phosphate buffered saline (PBS) for 5 min. The mice were then decapitated. The brain was dissected on ice, snap-frozen in liquid nitrogen, and stored at 80°C until time of use. Brain tissue (cortex or hippocampus) was homogenized using high shear homogenizer, in a 1:10 (w/v) of ice-cold freshly prepared lysis buffer consisting of PBS containing 1 μg/ml Leupeptin, 1 mM PMSF, and 1 mM EDTA. The tissue homogenate was centrifuged at 14,000 × *g* for 20 min at 4°C in a microcentrifuge, and supernatants were collected and stored at -80°C until use.

### Microglia and mixed glia cultures

Microglia cultures were prepared as previously described [32]. Briefly, mixed glial cultures (approximately 95% astrocytes, approximately 5% microglia) were prepared from the cerebral cortices of 1 to 3 day old mice. The tissue was trypsinized, and the cells were resuspended in glia complete medium (α-minimum essential medium (α-MEM; Mediatech, Manassas, VA, USA) supplemented with 10% fetal bovine serum (FBS) (US Characterized FBS; Hyclone; Cat no. SH30071.03), 100 IU/ml penicillin, 100 μg/ml streptomycin, and 2 mM L-Glutamine). After 10 to 14 days in culture, microglia were isolated from the mixed glial cultures by the shake-off procedure [33]. Loosely adherent microglia were shaken off at 250 rpm for 2 hr at 37°C. The cell-containing medium was centrifuged at 180 × *g* for 3 min, and the cells were seeded onto 48-well plate at a density of 2 × 10^4^. Alternatively, the mixed glia cultures were trypsinized and seeded onto a 48-well plate at a density of 2 × 10^4^. Cells were maintained for 24 hr in glia complete medium. LPS (3 ng/ml) or saline veh was then added directly to the glia complete medium for 30 min, after which the LPS- or veh-containing media was removed, the cells were washed once in glia complete medium, and then maintained for 24 hr in glia complete medium. Aliquots (5 μl) of the conditioned medium from the primary microglia and the mixed glia cultures were harvested at different times for cytokine measurements.

### Peritoneal macrophages and cortical microglia isolation for gene expression

Peritoneal macrophages were isolated following standard procedures as previously described [34,35]. Briefly, the mice were anesthetized, and 10 ml of PBS was injected into and recollected from the peritoneal cavity. The mice were then transcardiac-perfused with ice-cold PBS. The brain was harvested and the cortex was collected for microglia isolation. Microglia were isolated from the brain using a Percoll gradient following standard procedures as previously described [36]. Briefly, the brain tissue was homogenized in 6.5 ml of ice-cold PBS using a dounce homogenizer. The brain homogenate was added to 3 ml of 100% isotonic Percoll (ISP) in a 15-ml conical tube and mixed by inversion. A 2-ml layer of 50% ISP and 2-ml layer of 70% ISP were layered under the 100% ISP/brain homogenate (30% ISP in solution). The 15-ml conical tube containing the Percoll gradient was centrifuged at 500 × *g* for 20 min at room temperature. Microglia were collected at the 70% to 50% ISP interface. Peritoneal macrophages and cortical microglia were washed once in PBS before RNA extraction. RNA was isolated using an RNeasy kit (Qiagen, Valencia, CA, USA) according to the manufacturer’s protocols. RNA quantity and quality were determined using A260/A280 readings by NanoDrop (Thermo Scientific). Reverse transcription (RT) was done following the manufacturer's protocol using a High Capacity cDNA Reverse Transcription Kit (Applied Biosystems, Life Technologies, Grand Island, NY, USA). Controls included no template and no RT conditions. Real-time PCR was performed using the TaqMan Gene Expression Assay kit (Applied Biosystems, Life Technologies, Grand Island, NY, USA) according to the manufacturer's instructions on a ViiA™ 7 Real-Time PCR System (Applied Biosystems, Life Technologies, Grand Island, NY, USA). The following TaqMan probes (Applied Biosystems) were used: CCR2 (Mm00438270-m1); CX3CR1 (Mm02620111_s1); MAPK14 (Mm00442497_m1); 18S (Hs99999901_s1). Relative gene expression was calculated by the 2 - ^ΔΔCT^ method.

### Cytokine measurements

IL-1ra levels were measured with an R&D Systems DuoSet ELISA kit (R&D Systems, Minneapolis, MN, USA: cat. no. DY480) according to the manufacturer’s specifications. All other cytokine levels were measured as previously described [27,37] using Meso Scale Discovery (MSD: Rockville, MD, USA) custom multiplex high-sensitivity ELISA kits according to the manufacturer’s instructions. Tissue cytokine levels were normalized to the total amount of protein in the sample, as determined by BCA Protein Assay (Pierce, Rockford, IL, USA).

### Statistics

Statistical analysis was conducted using GraphPad prism software V.6 (GraphPad Software, La Jolla, CA, USA: http://www.graphpad.com). Comparisons between LPS-stimulated WT and p38α^ΔLysM-Cre^ KO mice were made by unpaired t-test. Comparisons were not made between naïve, or vehicle-injected mice as the cytokine levels were found to be consistently below or at the limit of detection. Statistical significance was defined as *P* <0.05. Values are expressed as mean ± SEM.

## Results

### The *in vitro* levels of TNFα in response to lipopolysaccharide are reduced in the p38α^ΔLysM-Cre^ knockout mice

We have previously demonstrated using primary microglia cultures that the induction of TNFα in response to TLR activation is governed by p38α, such that loss or inhibition of p38α results in less TNFα in response to stimuli [27,32]. However, *in vivo,* we recently found that the role of p38α in regulating proinflammatory cytokine responses, including TNFα, is more complicated than that predicted from our *in vitro* results. Specifically, using a LysM-Cre mouse line to selectively knock out p38α in cells of the myeloid lineage (p38α^ΔLysM-Cre^ KO mice), we found that the acute (6 hr post-injury) proinflammatory cytokine response to a diffuse TBI was enhanced in the KO mice compared to WT controls, even though the chronic cytokine levels were reduced in the p38α^ΔLysM-Cre^ KO mice [28]. These *in vivo* results suggest that p38α can both stimulate and unexpectedly limit the CNS proinflammatory cytokine response to injury. However, the mechanism by which p38α may suppress the acute CNS proinflammatory cytokine response has yet to be identified.

**Figure 1:**
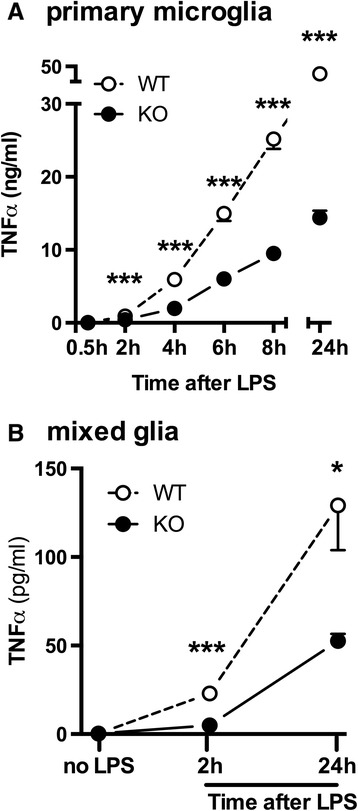
**Microglia and mixed glia cultures from p38α** ^**ΔLysM-Cre**^ **knockout (KO) are less responsive to lipopolysaccharide (LPS).** Wild-type (WT) (white) or p38α^ΔLysM-Cre^ KO (black) primary microglia **(A)** and mixed glia **(B)** were stimulated with LPS for 30 min, cells washed, and then conditioned media was harvested at select time points for TNFα Meso Scale Discovery (MSD) assays. The data are representative of three independent experiments. (*P <0.05; ***P <0.0005).

To begin to elucidate the mechanism by which p38α could limit the CNS proinflammatory cytokine response, we returned to our *in vitro* primary microglia and mixed glia assays. Our prior work measured the levels of TNFα that accumulated in the conditioned media with LPS present in cultures for a minimum of 18 hr. However, our *in vivo* TBI results showed that the acute cytokine response was higher in the p38α^ΔLysM-Cre^ KO mice at 6 hr post-injury, but lower by 7 d post-injury [28]. We hypothesized that p38α may have different effects temporally in the TNFα response (that is, an acute spike during the first 6 hr in TNFα levels), which we did not detect in our prior *in vitro* assays when LPS was continuously present, and TNFα was allowed to accumulate in the well for 18 hr. To test this idea, we treated primary microglia cultures with LPS for only 30 min, then removed the media, washed the cells once, and then added fresh medium (without LPS). The purpose of removing the LPS was to eliminate the chronic TLR4 pathway activation, which we know in microglia is partially dependent on p38α [27,32]; in this way we could look more selectively at feedback mechanism. We sampled 5 μl of microglia-conditioned media every 2 hr for the first 8 hr and at 24 hr after LPS stimulation, and measured TNFα levels. As shown in Figure 1A, at all time points from 2 to 24 hr, significantly lower TNFα levels were found in the p38α^ΔLysM-Cre^ KO primary microglia compared to the WT microglia (***P <0.0005), in agreement with our prior work [27,32].

The primary microglia assays we use are enriched such that approximately 95% of the cells are microglia. Therefore, we postulated that an interaction between astrocytes and microglia might account for the *in vivo* findings, which would not be observed when microglia were in isolation from astrocytes. To test this potential mechanism, we measured the production of TNFα in mixed glia cultures (astrocytes and microglia) treated with LPS and found that the levels of TNFα were also significantly lower in the mixed glia from p38α^ΔLysM-Cre^ KO mice compared to the WT mice (Figure 1B). Thus, primary microglia or mixed glia cultures derived from p38α^ΔLysM-Cre^ KO mice show significantly less TNFα induction in response to LPS stimulation compared to WT mice at all time points examined from 2 to 24 hr. Overall, these results do not explain the *in vivo* observation of enhanced acute proinflammatory cytokine response following TBI in p38α^ΔLysM-Cre^ KO mice.

### *In vivo,* p38α is efficiently deleted in acutely isolated adult peritoneal macrophages, but not in acutely isolated microglia from p38α^ΔLysM-Cre^ knockout mice

Recently, Butovsky *et al*. (2014) reported the gene expression signature of acutely isolated lymphocytes (B and T cells), myeloid cells (that is, dendritic cells, macrophages, and microglia), as well as astrocytes, oligodendrocytes, and neurons [38]. Microglia were found to express 10 times higher levels of p38α (MAPK14) compared to astrocytes, oligodendrocytes, or neurons. In addition, microglia expressed two- to threefold more p38α than other tissue macrophage populations [38]. We previously reported [27,32] that p38α is efficiently depleted in primary microglia from p38α^ΔLysM-Cre^ KO mice. However, the LysM-Cre mouse line may not have equal efficiency at knocking down p38α *in vivo* in all myeloid cell populations. For example, the recombination efficacy of the LysM-Cre in microglia in the adult brain has been shown to be between 20 and 45%, depending on the brain region and the method used to assess the recombination [39]. Since both the relative expression of p38α and the efficiency of the LysM-Cre promoter can vary in different myeloid cell populations, we measured the level of p38α suppression in acutely isolated peritoneal macrophages and cortical microglia from WT and p38α^ΔLysM-Cre^ KO mice.

**Figure 2:**
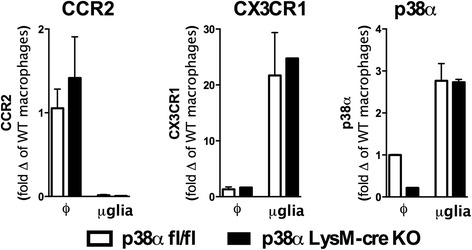
**Isolation of peritoneal macrophages (φ) and microglia (μglia) from adult mice shows enrichment of CCR2 and CX3CR1 cell populations, and loss of p38α in the isolated macrophages but not in the microglia fraction.** RNA was extracted from peritoneal macrophages or microglia isolated from cortical tissue using a discontinuous Percoll gradient. qRT-PCR using TaqMAN Gene Expression Assays was used to determine CCR2, CX3CR1, and p38α (MAPK14) expression in the isolated cell populations. Data are presented as fold change of wild-type (WT) macrophages. The data are representative of two independent experiments.

Using CCR2 and CX3CR1 as markers for macrophages and microglia, respectively, we found high levels of CCR2 in the peritoneal macrophages, and high levels of CX3CR1 in the microglia cell fraction (Figure 2), consistent with previous studies [40]. The gene expression of CCR2 and CX3CR1 indicated that our isolation resulted in enrichment of the target cell populations. In the WT mice, we confirmed that expression of p38α was higher in the microglia fraction compared to the macrophage population (Figure 2), as previously reported [38]. Interestingly, in the p38α^ΔLysM-Cre^ KO mice, macrophages but not microglia were found to have efficient deletion of p38α (Figure 2). It should be noted that CCR2 and CX3CR1 expression alone does not indicate whether cells other than macrophages or microglia are present in our cell fractions; therefore these results may underestimate the percentage of microglia that may be p38α deficient.

These findings would appear to be in disagreement with our previous reports that p38α is efficiently depleted in primary microglia from p38α^ΔLysM-Cre^ KO mice [27,32]. One potential explanation for the discrepancy is the culture conditions, as primary microglia are activated by removal from the CNS tissue environment. The *in vitro* activation associated with the primary culture could be sufficient to increase the expression of LysM, and the 10 to 14 d of culture would be enough time for the Cre/loxP recombination to result in a loss of p38α. Therefore, microglia in culture appear to be more similar to peritoneal macrophages than to acutely isolated microglia from adult mice in terms of efficiency of p38α knockdown.

### The proinflammatory cytokine response to intracerebroventricular (ICV) injection of LPS is not suppressed in the p38α^ΔLysM-Cre^ KO mice

**Figure 3:**
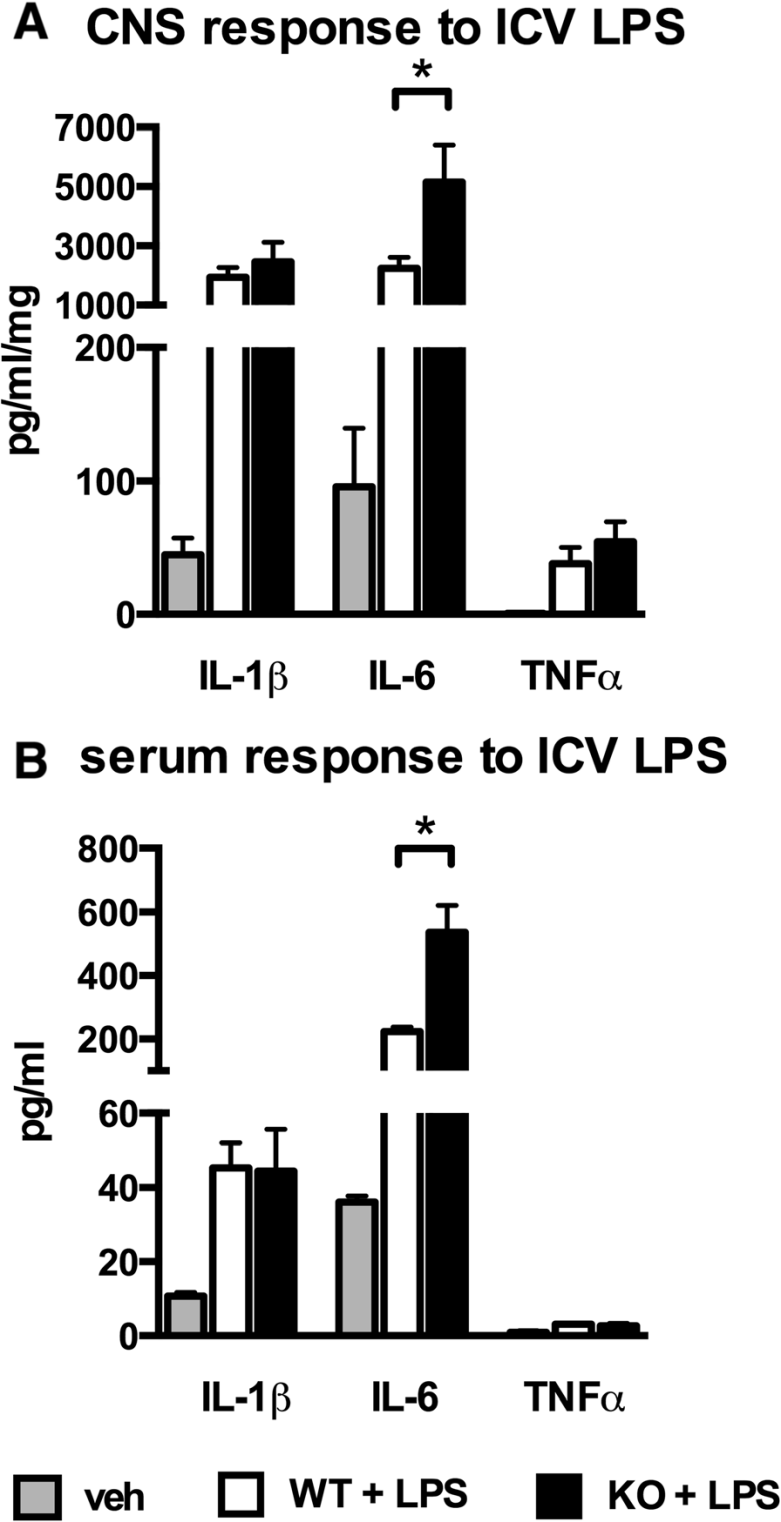
**p38α** ^**ΔLysM-Cre**^ **knockout (KO) mice show no suppression of the proinflammatory cytokine response to intracerebroventricular (ICV) injection of lipopolysaccharide (LPS).** At 6 hr post-ICV injection, levels of cytokines were measured in the ipsilateral hippocampus **(A)** and serum **(B)** of wild-type (WT) mice (white) or p38α^ΔLysM-Cre^ KO mice administered saline (vehicle (veh): gray) or 25 ng LPS (black). (n = 4 per veh group (WT = 2, KO = 2); n = 8 per LPS group) (*P <0.05).

We next postulated that the difference in the acute proinflammatory cytokine response between our *in vivo* diffuse TBI model and our *in vitro* LPS model could be the result of different stressor stimuli. To test this idea, we induced a sterile inflammatory response in the CNS by the injection of 25 ng of LPS into the right lateral ventricle of WT mice and p38α^ΔLysM-Cre^ KO mice, thus mirroring our *in vitro* LPS assays. Mice were euthanized at 6 hr post-injection, which was determined by a preliminary experiment (data not shown) as the peak of the IL-1β response in the ipsilateral hippocampus following ICV LPS. Figure 3A shows that levels of three proinflammatory cytokines (IL-1β, IL-6, and TNFα) were increased in the hippocampus at 6 hr after ICV LPS administration. IL-1β and TNFα showed no difference between the LPS-stimulated WT and p38α^ΔLysM-Cre^ KO mice. The only difference between LPS-stimulated WT and p38α^ΔLysM-Cre^ KO mice in CNS responses was a significantly higher up-regulation of IL-6 (t_(14)_ =2.246 p =0.014) in the cortex, as well as in the serum of p38α^ΔLysM-Cre^ KO mice compared to WT mice (Figure 3B: (t_(14)_ =3.635 p =0.0027)). We replicated this experiment twice with 100 ng and 1 μg of LPS and found no difference in IL-1β levels between p38α^ΔLysM-Cre^ KO mice compared to WT mice at 6 hr post-injection (data not shown), indicating that the LPS dose was not a critical variable. We previously found that IL-1β, IL-6 and TNFα were increased both at the protein and mRNA level in the cortex following a diffuse TBI in p38α^ΔLysM-Cre^ KO mice [28]. Therefore, the ICV LPS model more closely resembled our *in vivo* diffuse TBI model, than our *in vitro* LPS model. However, the ICV LPS model did not mirror the overproduction of IL-1β and TNFα seen in the *in vivo* diffuse TBI model. Nevertheless, the ICV LPS model did not show a reduced proinflammatory cytokine response in the p38α^ΔLysM-Cre^ KO mice, as seen in the *in vitro* LPS model. A difference in the stressor stimuli is thus not able to explain the acutely elevated levels of proinflammatory cytokines in the CNS following diffuse TBI in p38α^ΔLysM-Cre^ KO mice [28]. As microglia in the p38α^ΔLysM-Cre^ KO mice are not p38α deficient *in vivo*, the overproduction of IL-6 in response to ICV LPS may come from systemic cells, or circulating factors.

### The CNS proinflammatory cytokine response to systemic lipopolysaccharide is enhanced, while the systemic proinflammatory response is suppressed in the p38α^ΔLysM-Cre^ knockout mice

**Figure 4:**
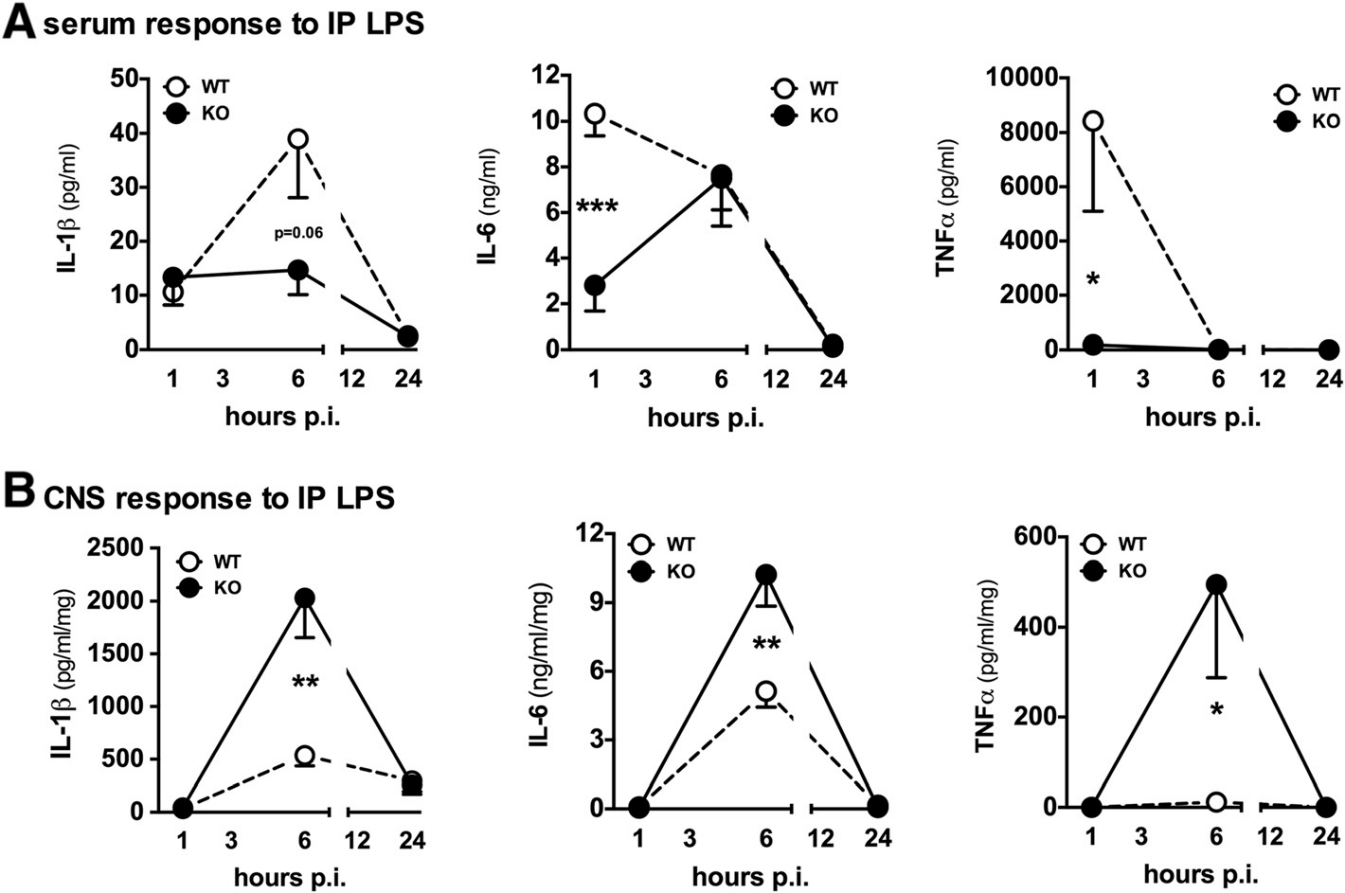
**Levels of pro-inflammatory cytokines in the serum and cortex of mice following systemic lipopolysaccharide (LPS) stimulation.** Wild-type (WT) mice (white) or p38α^ΔLysM-Cre^ knockout (KO) mice (black) were injected intraperitoneally (IP) with 5 mg/kg LPS. The cytokine response in the serum was significantly reduced in the p38α^ΔLysM-Cre^ KO mice **(A)**, while the response in the CNS was increased in the p38α^ΔLysM-Cre^ KO mice compared to the WT mice **(B)** (n =5 to 8 per group) (*P <0.05; **P < 0.01; ***P <0.001).

To test the hypothesis that an altered systemic inflammatory response may account for the increased proinflammatory cytokine response in the CNS following diffuse TBI in p38α^ΔLysM-Cre^ KO mice, we used an intraperitoneal (IP) LPS model. WT and p38α^ΔLysM-Cre^ KO mice were injected IP with 5 mg/kg LPS (3 × 10^6^ EU/mg). Additionally, to further explore differences in the temporal patterning of the proinflammatory cytokine response in the WT and p38α^ΔLysM-Cre^ KO mice, serum and cortical tissue were harvested at different time points after LPS insult. In agreement with previous studies [29], we found a significantly lower serum cytokine response to LPS in the p38α^ΔLysM-Cre^ KO mice compared to WT mice (Figure 4A). At 1 hr post-injection, the p38α^ΔLysM-Cre^ KO mice had significantly less IL-6 (t_(8)_ =5.062 *P* = 0.001) and TNFα (t_(8)_ =2.479 *P* =0.0382) compared to the WT mice. In agreement with our previous study in the diffuse TBI model [28], we found a significantly elevated peak LPS-induced CNS cytokine response in the p38α^ΔLysM-Cre^ KO mice compared to the WT mice (Figure 4B). At 6 hr post-injection, levels of IL-1β (t_(16)_ = 3.823 *P* = 0.0015), IL-6 (t_(7)_ = 3.526 *P* = 0.0097), and TNFα (t_(16)_ = 2.328 *P* = 0.0333) were significantly higher in the p38α^ΔLysM-Cre^ KO compared to the WT mice. In an independent experiment, we used a lower dose of LPS (1 mg/kg) and still found a significant increase in IL-1β and IL-6 in the CNS of p38α^ΔLysM-Cre^ KO mice compared to the WT mice at 6 hr post injection (data not shown). Therefore, the results of the IP LPS model support the hypothesis that systemic cells, or circulating factors dependent on p38α, are important for limiting the proinflammatory cytokine response in the CNS.

### The p38α^ΔLysM-Cre^ knockout mice fail to produce IL-10 in response to systemic lipopolysaccharide

**Figure 5:**
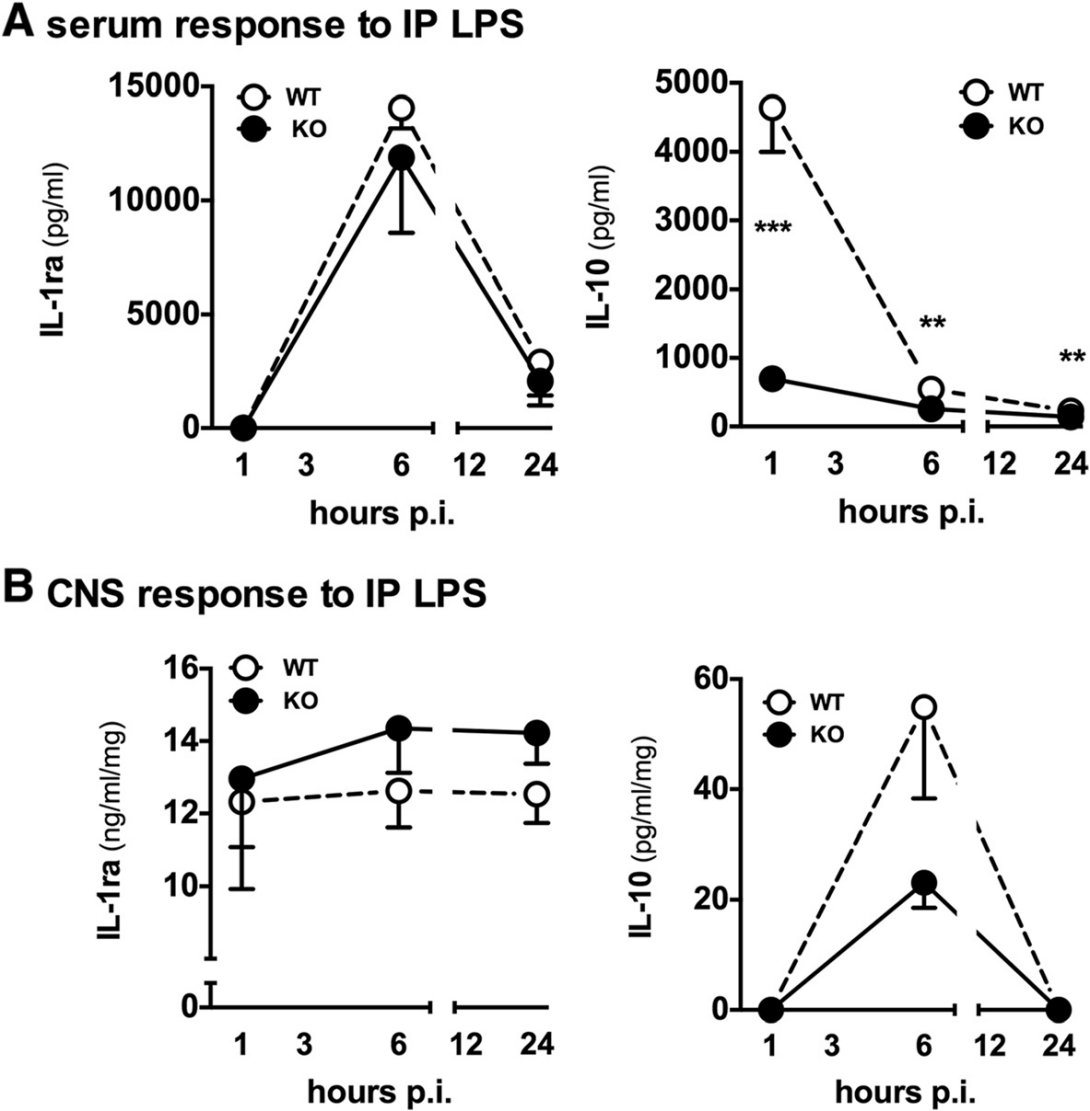
**Levels of anti-inflammatory cytokines in the serum and cortex of mice following systemic lipopolysaccharide (LPS) stimulation.** Wild-type (WT) mice (white) or p38α^ΔLysM-Cre^ knockout (KO) mice (black) were injected intraperitoneally (IP) with 5 mg/kg LPS. No significant differences in IL-1ra levels in serum **(A)** or CNS **(B)** were detected between WT and p38α^ΔLysM-Cre^ KO mice. Levels of IL-10 in the serum were significantly reduced in the p38α^ΔLysM-Cre^ KO mice **(A)**, and there was a trend for reduced levels of IL-10 in the CNS **(B)** although the data did not reach statistical significance (n =5 to 8 per group) (**P <0.01; ***P <0.001).

It has been previously reported that p38α is involved in the production of two anti-inflammatory cytokines, IL-1ra [41] and IL-10 [42,43]. Therefore, we hypothesized that loss of a paracrine anti-inflammatory response might be responsible for the increased CNS proinflammatory cytokines in the p38α^ΔLysM-Cre^ KO mice. To test this, we measured serum and cortex levels of IL-1ra and IL-10 after systemic LPS administration to WT and p38α^ΔLysM-Cre^ KO mice. We found no significant difference in the levels of IL-1ra in the serum between WT and p38α^ΔLysM-Cre^ KO mice at any time point measured (Figure 5A). In contrast, we found that the levels of IL-10 in the serum were markedly suppressed in the p38α^ΔLysM-Cre^ KO mice compared to the WT mice following LPS stimulation (Figure 5A). At 1 hr, a highly significant (t_(8)_ = 6.033 *P* = 0.0003) suppression of the IL-10 response was seen in the p38α KO mice. The levels of IL-10 in the serum of the p38α^ΔLysM-Cre^ KO mice remained suppressed at the 6 hr (t_(8)_ = 4.996 *P* = 0.0011) and 24 hr (t_(8)_ = 3.727 *P* = 0.0058) time points compared to the WT mice. In the cortex, there was also no significant difference in the levels of IL-1ra (Figure 5B) between p38α^ΔLysM-Cre^ KO and WT mice. At 6 hr after LPS, there were lower IL-10 levels in the cortex of p38α^ΔLysM-Cre^ KO mice compared to the WT mice, but the decrease did not reach significance (Figure 5B). Thus, a loss of the systemic IL-10 response in the p38α^ΔLysM-Cre^ KO could be the mechanism by which proinflammatory cytokine response in the CNS becomes exaggerated.

## Discussion

The goal of this project was to explore the potential mechanism by which p38α could suppress the acute proinflammatory cytokine response in the CNS. Our prior work, using *in vitro* assays, demonstrated that p38α is critical for the production of IL-1β and TNFα from activated microglia [27,32]. We also previously showed, using a small molecule p38α inhibitor, that suppression of the p38α signal transduction pathway limits the IL-1β response in the CNS to systemic LPS [17,27,44]. Moreover, we found that p38β MAPK is not involved in the CNS cytokine production, or in neurotoxicity induced by LPS inflammatory insult or multiple toxic insults [45,46]. Despite this strong precedent for p38α, we recently found that in a diffuse TBI model the acute proinflammatory cytokine response in the CNS was higher in the p38α^ΔLysM-Cre^ KO mice [28]. Therefore, we sought to determine the mechanism by which p38α could limit the acute proinflammatory response, as this could be critical in the use of p38α as a therapeutic target to treat diseases of the CNS that have inflammation as one component of the pathophysiology.

**Figure 6:**
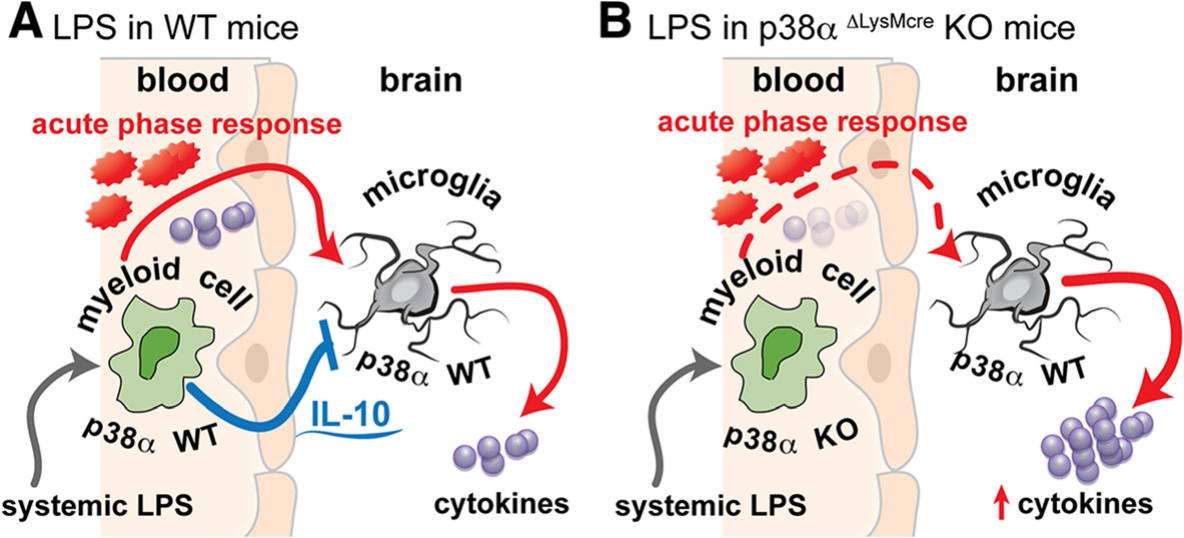
**Proposed model for p38α in myeloid cell cytokine response to systemic lipopolysaccharide (LPS). (A)** In wild-type (WT) mice, myeloid cells respond to systemic LPS by producing an acute phase response, including the production of proinflammatory cytokines, as well as the anti-inflammatory cytokine IL-10. The systemic response spreads to the brain where microglia produce cytokines locally in the CNS. **(B)** In the p38α^ΔLysM-Cre^ knockout (KO) mice, the systemic acute phase response is suppressed such that there is less proinflammatory cytokine production in the blood. However, there is also a loss of the negative feedback provided by the anti-inflammatory IL-10, which we propose leads to an exaggerated acute proinflammatory cytokine response in the CNS.

Collectively, our results suggest that p38α has a critical role in limiting the acute cytokine storm, and the spread of the systemic innate immune response to the CNS, by regulating the production of the anti-inflammatory cytokine IL-10. A peripheral injection of LPS into the peritoneal cavity initiates a systemic innate immune response. There remains a debate of how the peripheral inflammation leads to a neuroinflammatory response. However, acute phase proteins (that is, prostaglandin E2), cytokines (that is, IL-1β) and LPS itself have been suggested to be involved in the mechanism by which a peripheral LPS stimulation induces neuroinflammation [47-51]. In agreement with previous studies [29], we found that levels of the proinflammatory cytokines IL-1β, IL-6, and TNFα were all decreased in the serum of mice where macrophages were deficient in p38α. Despite the decrease in the proinflammatory cytokine response in the serum, the peak of the proinflammatory response in the CNS was significantly higher in the p38α^ΔLysM-Cre^ KO mice. Our data are consistent with a model (Figure 6) whereby systemic LPS induces an acute phase response, including upregulation of both pro- and anti-inflammatory cytokines. In WT mice, the balance of the pro- and anti-inflammatory cytokine response, along with other acute phase mechanisms, leads to an increase in cytokines in the CNS (Figure 6A). However, if circulating IL-10 levels are not elevated to counterbalance the increased systemic proinflammatory responses, the spread of the inflammatory response from the periphery to the CNS is exaggerated. This model is also consistent with data from IL-10 deficient mice, in a large number of *in vivo* inflammatory models, which collectively suggest that no other anti-inflammatory cytokine can compensate for the loss of IL-10, because without IL-10, there will be aberrant proinflammatory cytokine overproduction (for review see: [52]). Our results reported here demonstrate that p38α is required by myeloid cells to produce IL-10 in response to systemic LPS, and without an effective IL-10 response - a defect seen in the p38α^ΔLysM-Cre^ KO mouse - neuroinflammation can be exaggerated (Figure 6B).

The systemic balance between pro- and anti-inflammatory factors can influence the proinflammatory cytokine response in the CNS. For example, relevant to the current study, IL-10 is able to block proinflammatory cytokine upregulation in the CNS in response to ICV or IP LPS administration [53,54] and to spinal cord injury [55]. Moreover, IP LPS in IL-10 KO mice results in a significantly exaggerated TNFα, IL-1β and IL-6 response in the plasma and in the CNS compared to WT mice [56], following a similar pattern to our results. Both microglia and astrocytes express the IL-10 receptor; however, *in vitro* IL-10 was found to have a suppressive effect only on astrocytes and not microglia [57]. The effect of IL-10 on microglia was found to be indirect through the astrocyte production of TGFβ [57]. We do not know if this mechanism would also be true in our model, but understanding the crosstalk between microglia and astrocytes in regulating the balance between pro- and anti-inflammatory factors is an important future direction.

The broad anti-inflammatory properties of IL-10 are a result of IL-10’s ability to inhibit innate function of macrophages, neutrophils, and dendritic cells including their production of proinflammatory cytokines [52,58]. The present study confirmed previous observations [43,59] that macrophages require p38α for the production of IL-10, which suggests that the induction of this anti-inflammatory cytokine may limit the CNS inflammatory response to LPS. This finding is in agreement with the role of mitogen-and-stress-activated kinases 1 and 2 (MSK1/2) in the production of IL-10 [42,43]. MSK1/2 is activated by p38 and Erk1/2 [60]. Microglia and macrophages deficient in p38α have been shown to have decreased levels of the activated phospho-MSK1/2 [27,42,43]. Critically, MSK1/2 double KO mice are found to have elevated proinflammatory cytokine responses to LPS, which is IL-10 dependent, as illustrated elegantly by the use of an MSK1/2 / IL-10 triple KO mouse [42]. In addition to the MSK1/2 pathway, p38α can also regulate IL-10 expression in macrophages via the direct downstream target of p38α, the MAPK-activated protein kinase 2 [61]. Defining why Erk1/2 is not able to compensate for the loss of p38α will require future studies to understand potential crosstalk between p38α and Erk1/2 in regulating IL-10 levels following *in vivo* LPS stimulation.

## Conclusions

We and others have previously reported using small molecule inhibitors that suppression of p38α can reduce neuroinflammation *in vivo* [16,17,19,23,27]. In the current study, we found that the peak proinflammatory cytokine response to systemic LPS *in vivo* is significantly greater in the CNS of p38α^ΔLysM-Cre^ KO mice compared to the WT mice, replicating the result found in a diffuse TBI model [28]. We also found that after systemic stimulation of the innate immune system with LPS, p38α was necessary for a stimulus-induced increase in serum levels of the anti-inflammatory cytokine IL-10. Therefore, p38α appears to be a fulcrum in balancing the systemic innate immune response, by regulating levels of pro- and anti-inflammatory cytokines. While p38α appears to be a promising therapeutic target, our work here highlights the complexity of such efforts. The observations that p38α may balance the inflammatory response by acutely attenuating the early proinflammatory cytokine surge, while perpetuating the chronic microglia activation response raise the importance of considerations of an optimal therapeutic window for the efficacy of p38α inhibitors. Our findings that p38α-dependent systemic inflammatory responses affect the CNS inflammatory responses also suggest that measurement of systemic IL-10 and TNFα levels in serum or plasma might be a useful tool for monitoring the anti- and pro-inflammatory cytokine balance during the course of treatment of CNS disorders.

## Abbreviations

AD: Alzheimer's disease
CNS: central nervous system
ICV: intracerebroventricular
IP: intraperitoneal
IL-1: interleukin-1
IL-1ra: IL-1 receptor antagonist
ISP: isotonic Percoll
KO: knockout
LPS: lipopolysaccharide
MAPK: mitogen-activated protein kinase
MSD: Meso Scale Discovery
MSK1/2: mitogen-and-stress-activated kinases 1 and 2
RT: reverse transcription
TLR: toll-like receptor
TNFα: tumor necrosis factor alpha
veh: vehicle
WT: wild type
